# Risk factors for endometrial carcinoma among postmenopausal women in Sri Lanka: a case control study

**DOI:** 10.1186/s12889-019-7757-2

**Published:** 2019-10-28

**Authors:** Withanage Iresha udayangani Jayawickcrama, Chrishantha Abeysena

**Affiliations:** 1 Postgraduate Institute of Medicine, Colombo, Sri Lanka; 2 Department of Public Health, Faculty of Medicine, University of Kelaniya, Ragama, Sri Lanka

**Keywords:** Endometrial carcinoma, Risk factors, Postmenopausal women

## Abstract

**Background:**

Endometrial carcinoma burden is on the rise globally. The objective of this study was to determine the risk factors for endometrial carcinoma among postmenopausal women in Western province in Sri Lanka.

**Methods:**

A case control study was conducted recruiting 83 incident cases of endometrial carcinoma and 332 unmatched hospital controls from all the secondary and tertiary care hospitals in the province using consecutive sampling technique. A case was defined as a postmenopausal woman who had been residing in the province for at least a period of 1 year, diagnosed to have endometrial carcinoma with histological confirmation within 3 months of the initiation of data collection of the study. Data were collected using validated interviewer administered questionnaire. Risk factor were identified through multiple logistic regression and results were expressed as adjusted odds ratios (AOR) and 95% confidence intervals (CI).

**Results:**

The independent risk factors of endometrial carcinoma are having family history of any type of cancer among first degree relative (AOR = 12.6; 95% CI:5.14–30.9), generalized obesity (BMI ≥25 kg/m^2^) (AOR = 11.85; 95% CI:5.12–27.4), never conceived (AOR = 3.84; 95% CI:1.37–10.7), age at menarche ≤11 years (AOR = 4.07; 95% CI:1.16–14.2), age > 55 years (AOR = 4.69; 95% CI:2.16–10.2), monthly family income of ≤20,000 Rupees (AOR = 2.65; 95% CI:1.31–5.39), sub-optimal consumption of deep fried food (AOR = 0.17; 95% CI:0.06–0.46), and low level household activities (AOR = 2.82; 95% CI:1.34–5.92).

**Conclusions:**

There were eight independent risk factors of endometrial carcinoma specific for Sri Lankan postmenopausal women identified. Some modifiable risk factors such as generalized obesity, sub-optimal dietary practices and low level physical activities need to be addressed at primary prevention level.

## Background

Endometrial cancer (EC) represents the most frequent gynaecological malignancy in the developed countries, with an annual incidence of 19.5 cases per 100,000 women [1]. There were 320,000 new endometrial carcinoma cases diagnosed worldwide in 2012, accounting 4.8% of cancers among women [2]. According to the American Cancer Society [3], the probability of a woman to develop endometrial carcinoma during lifetime is 1 in 37. Population aging and rise in obesity both have joint impact on the rising incidence of endometrial carcinoma in developing countries, as evident by 2.8 times increase of age-standardized incidence rate from 3.02 cases per 100,000 women in 1991 to 11.36 cases per 100,000 women in 2010 in Taiwan [4]. When considering mortality, there were 76,000 deaths occurred due to endometrial carcinoma accounting 2.1% of cancer deaths among women, witnessing the good prognosis of the disease unless diagnosed at late stage. The five-year survival is almost 82% among the women who are diagnosed at early stages [2].

According to the latest statistics available in Sri Lanka, 4% of all reported incident cancer cases among women in 2010 were due to endometrial carcinoma. It was the eighth commonest cancer among women in Sri Lanka [5]. When the age group cancer distribution was compared, endometrial carcinoma was responsible for 6.4% of all the cancers seen among women of 50–64 years, and was the fourth commonest cancer among women. Furthermore, the rising incidence of the endometrial carcinoma is evident by the increased crude incidence rate from 2.6 per 100,000 women in 2007 to 4.5 per 100,000 women in 2014, showing almost a 1.7-fold increase [5]. As for mortality, endometrial carcinoma accounted for a crude death rate of 2.6 per 100,000 population in 2010 [5]. Increasing burden of disease morbidity worldwide, despite its low mortality in the early stage of disease still causes a huge socio-economic impact at individual, household and country level through direct and indirect costs.

Generally, the aetiology of endometrial carcinoma is multi factorial [6]. They are broadly categorized into non-modifiable and modifiable risk factor. Non-modifiable risk factors are age, race and genetic predisposition, while the modifiable life style factors are unhealthy diet and physical inactivity [7]. Further, nulliparity, obesity, diabetes, hypertension and hormone replacement therapy (HRT) are also shown as modifiable risk factors to develop endometrial carcinoma [8]. endometrial carcinoma risk is positively associated with early age at menarche, older age at menopause and use of oestrogen replacement therapy [6]. The knowledge gained by the risk factor studies conducted in Western and other Asian countries may not be directly applicable to Sri Lankan setting as the risk profile of Sri Lankan women may be different from them due to the diversity seen in social and cultural background. Hence, identifying country specific risk factors for endometrial carcinoma among Sri Lankan women would help the stake holders to plan and implement preventive programmes to reduce the magnitude of risk factors. These facts emphasize the need of identifying population-specific risk factors for endometrial carcinoma among Sri Lankan women. Therefore, the objective of this study was to determine the risk factors for endometrial carcinoma among postmenopausal women in Western province in Sri Lanka.

## Methods

A hospital based unmatched case-control study was done from September 2016 to March 2017. The cases and controls were selected from the secondary and tertiary care state hospitals in the Western province where a Consultant Gynaecologist and a Consultant Pathologist were available. These hospitals were expected to represent all the patients diagnosed with endometrial carcinoma in the government sector. Western province is one of the nine provinces in Sri Lanka which includes the largest share of the population of Sri Lanka (28.7%) [9]. It is divided into three administrative districts namely, Colombo, Gampaha and Kalutara. It consists of one National Hospital, nine Teaching Hospitals, three District General Hospitals, four Base Hospitals, 44 Medical Officer of Health areas and many Primary Health Care Centres to provide curative, palliative and preventive health services under the state sector.

A case was defined as a postmenopausal woman who had been residing in the Western province for at least a period of 1 year, diagnosed to have endometrial carcinoma with histological confirmation within 3 months of the initiation of data collection of the study. Patients with recurrence of endometrial carcinoma, secondary endometrial carcinoma, diagnosed of any other cancer except breast cancer patients who were treated with Tamoxifen and patients who had been admitted to intensive care or high dependency unit care were excluded. The cases of endometrial carcinoma were recruited from the Oncology and Gynaecology wards and clinics. A control was defined as a postmenopausal woman who had been residing in the province for at least one-year duration prior to the commencement of the study, and confirmed as not having endometrial carcinoma by performing hysteroscopy, endometrial biopsy or curettage within 3 months of the initiation of data collection of the study. Ideally, the controls should be healthy women selected from the community where the cases belonged, since the controls should be comparable to the source population of the cases. Though, it is unethical to carry out invasive procedures to healthy women to exclude any cancer status at the time of selection. Therefore, in this study, controls were also selected from Gynaecology wards and clinics of all 14 hospitals which the cases were selected to reduce the selection bias.

The required sample size was calculated for several common risk factors of endometrial carcinoma and the largest calculated value was selected as the required sample size for the study. The values for odds ratios (OR) for different risk factors, their community prevalence were based on the available literature. The corresponding risk factor for endometrial carcinoma was obesity with an odd ratio of 2.51 and 11% of the Sri Lankan women were obese [10, 11]. Case to control ratio was taken as 1:4. For the calculation of the sample size, power was taken as 80%. The largest sample size calculated was 79 and adding 5% for non-response, the total required sample size for the cases was 83. Therefore, the required number of controls was 332.

An interviewer administered questionnaire was used to assess potential risk factors and it was developed based on the evidence taken through thorough literature review (Additional file 1). Quality of diet and physical activity related to cancer were assessed by validated Food Frequency Questionnaire^12^ (Additional file 2) and Lifetime Total Physical Activity Questionnaire [12] (Additional file 3). Further refining of the questions following the initial development of the questionnaire was carried out by conducting consultative meetings with the experts from the field of Community Medicine, Gynaecology and Oncology. The discussions were continued till there was consensus on the questionnaire by the group of experts. The questionnaires were pre-tested among 20 post- postmenopausal women. Face and content validity of the questionnaire was assessed. Five pre-intern medical officers were selected and trained as data collectors. Written consent was taken prior to the administration of the questionnaire. Steps were undertaken to minimize the disturbances to the routine work of the wards and the clinics.

Quality of diet was assessed into optimal or sub-optimal consumption of energy dense food, food containing dietary fibre and anti-oxidants using validated Food Frequency Questionnaire [12]. Overconsumption of energy dense food and inadequate consumption of food containing dietary fibre and anti-oxidants were considered as sub-optimal consumption. Physical activity was defined as life time total physical activity in terms of occupational, household, sports and exercise activities and assessed by calculating the average Metabolic Equivalent of Task (MET) value for total physical activities for a week during a year by validated Life Time Total Physical Activity Questionnaire [12]. The Metabolic Equivalent values less than 25th percentile was considered as low, more than 75th percentile was high and in between 25th to 75th percentile was taken as average physical activity in life. Exposure to electromagnetic field was defined as a distance less than 100 m from home to high tension wires [13, 14] and telecommunication towers [15]. Exposure to outdoor air pollution was defined as having the house within 100 m from main road or having a large industry within one km [16]. Long term illness was defined as diagnosis of diabetes mellitus, hypertension and hyperlipidaemia [17]. Generalized obesity was categorized based on Body Mass Index (BMI) according to the World Health Organization (WHO) definitions for adult Asians, Body Mass Index value of 25 kg/m^2^ or more was categorized as obese. Central obese was defined as waist circumference of 80 cm or more based on American Diabetes Association Criteria.

Data analysis was done using the Statistical Package for the Social Sciences (SPSS)-16th version. A bivariate analysis was carried out to assess the association of each variable with the endometrial carcinoma. A multiple logistic regression was performed to identify the independent risk of each variable with endometrial carcinoma. The variables included in the regression model were the variables that showed *p*-value of < 0.2 in the bivariate analysis. The purposeful selection method was employed. Goodness of fit of the model was assessed by Hosmer and Lameshow test which was satisfactory (*p* = 0.99). There was no evidence of interaction and multicollinearity.

Ethical clearance to carry out the study was obtained from the Ethics Review Committee, Faculty of Medicine, University of Kelaniya, Sri Lanka.

## Results

The study sample consisted of 83 patients diagnosed with endometrial carcinoma and 332 controls diagnosed with endometrial atrophy, endometrial prolapse, endometritis and atrophic vaginitis at the time of recruitment. The response rate of the cases and controls were 100%.

A great majority of the cases were recruited from the hospitals located in Colombo district (97.6%, *n* = 81). The highest proportion of cases in the Colombo district was recruited from National Cancer Institute, Maharagama (90.4%; *n* = 75). Most of the controls were also recruited from the hospitals located in Colombo district (61.3%; *n* = 203), while 25.5% (*n* = 85) from the hospitals located in Gampaha district and 13.2% (*n* = 44) from the hospitals located in Kalutara district. The highest proportion of cases belonged to the age category of 60–69 years (*n* = 35; 42.2%) while the highest proportion of controls belonged to the age category of 50–59 years (*n* = 126; 38.0%). The median age of cases and controls were 61.0 (IQR = 56.0–67.0) years and 55.0 (IQR = 50.0–65.0) years respectively.

There was a higher proportion of age > 55 years (*p* < 0.001), monthly family income of ≤20,000 Sri Lanka Rupees (*p* = 0.03), unmarried (*p* = 0.01), never conceived (*p* < 0.001), nulliparity (*p* < 0.001) and menarche age ≤ 11 years (*p* < 0.001) in the case group as compared with those of the control group (Table 1).

**Table 1:** Risk of Endometrial Carcinoma associated with Socio-Demographic Factors

Characteristic	Disease Status				OR	95% CI	*p* value
	Cases (*N* = 83)		Controls (*N* = 332)				
	n	%	n	%			
Age							
> 55 years	64	77.1	159	47.9	3.66	2.10–6.38	*p* < 0.001
≤ 55 years	19	22.9	173	52.1	1.0		
Ethnicity							
Sinhalese	73	87.9	291	87.6	1.03	0.49–2.15	*p* = 0.94
Non-Sinhalese^a^	10	12.1	41	12.4	1.0		
Religion							
Buddhist	68	81.9	265	79.8	1.15	0.62–2.13	*p* = 0.66
Non-Buddhist^b^	15	18.1	67	20.2	1.0		
Level of education							
A/Level and above	19	22.9	57	17.2	1.43	0.80–2.57	*p* = 0.23
Below A/Level	64	77.1	275	82.8	1.0		
Employment status							
Currently employed^c^	5	6.0	60	18.1	1.18	0.73–1.92	*p* = 0.49
Previously employed^c^	40	48.2	106	31.9	1.0		
Never employed	38	45.8	166	50.0			
Monthly Family Income (SLR)							
≤ 20,000	46	55.4	140	42.2	1.71	1.05–2.77	*p* = 0.03
> 20,000	37	44.6	192	57.8	1.0		

^a^Tamil/Muslim/Burgher combined

^b^Catholic/Christian/Islam/Hindu combined

^c^Previously employed/currently employed combined

There was a higher proportion of unmarried (*p* = 0.01), never conceived (*p* < 0.001), nulliparity (*p* < 0.001) and menarche age ≤ 11 years (*p* < 0.001) in the case group as compared with those of the control group (Table 2).

**Table 2:** Risk of Endometrial Carcinoma associated with Reproductive Factors

Characteristic	Disease Status				OR	95% CI	*p* value
	Cases (*N* = 83)		Controls (*N* = 332)				
	n	%	n	%			
Marital status							
Unmarried	8	9.6	11	3.3	3.11	1.21–8.01	*p* = 0.01
Ever Married	75	90.4	321	96.7	1.0		
History of conception							
Never conceived	17	20.5	24	7.2	3.31	1.68–6.50	*p* < 0.001
Ever conceived	66	79.5	308	92.8	1.0		
Parity							
Nulliparous	19	22.9	28	8.4	3.22		
3 or less^a^	48	57.8	245	73.8			
More than 3^a^	16	19.3	59	17.8	1.0	1.69–6.12	*p* < 0.001
Age at menarche							
≤ 11 years	11	13.3	10	3.0	4.92	2.01–12.02	*p* < 0.001
> 11 years	72	86.7	322	97.0	1.0		
Age at menopause							
> 55 years	4	4.8	5	1.5	3.31	0.87–12.61	*p* = 0.06
≤ 55 years	79	95.2	327	98.5	1.0		
Use of Hormone Replacement Therapy							
(HRT)							
Ever used	1	1.2	6	1.8	0.66	0.08–5.58	*p* = 0.70
Never used	82	98.8	326	98.2	1.0		
Use of Hormonal contraceptives							
Ever used	12	14.5	58	17.5	0.80	0.40–1.56	*p* = 0.51
Never used	71	85.5	273	82.5	1.0		
Total	83	100.0	332	100.0			

^a^3 or less/> 3 combined

There was a higher proportion of women with generalized obesity and central obesity (*p* < 0.001), and exposure to passive smoking (*p* = 0.01) in the case group as compared with those of the control group (Table 3).

**Table 3:** Risk of Endometrial Carcinoma associated with Life Style Related Factors

Characteristics	Disease Status				OR	95% CI	*p* value
	Cases (*N* = 83)		Controls (*N* = 332)				
	n	%	n	%			
Dietary fibre							
Sub-optimal^a^	48	57.8	172	51.8	1.28	0.78–2.07	*p* = 0.32
Optimal	35	42.2	160	48.2	1.0		
Anti-oxidants							
Sub-optimal^a^	41	49.4	196	59.0	0.67	0.42–1.10	*p* = 0.11
Optimal	42	50.6	136	41.0	1.0		
Energy dense food							
Sub-optimal^a^	2	2.4	27	8.1	0.28	0.06–1.20	*p* = 0.06
Optimal	81	97.6	305	91.9	1.0		
Lifetime total physical activity (MET hours per week per year)							
Low (< 68.86)	25	30.1	79	23.8	1.38	0.81–2.35	*p* = 0.23
Average (68.86–144.51)^b^	45	54.2	163	49.1			
High (> 144.51)^b^	13	15.7	90	27.1	1.0		
Occupational activities							
Low (< 1.00)	38	45.8	161	48.5	0.89	0.55–1.45	*p* = 0.66
Average (1.00–72.25)^c^	23	27.7	90	27.1			
High (> 72.25)^c^	22	26.5	81	24.4	1.0		
Household activities							
Low (< 56.54)	29	34.9	75	22.6	1.84	1.09–3.09	*p* = 0.02
Average (56.54–84.15)^d^	48	57.8	160	48.2			
High (> 84.15)^d^	6	7.2	97	29.2	1.0		
Sports and exercise activities							
Low (<1.00)	52	62.7	167	50.3	1.65	1.01–2.71	*p* = 0.04
Average (1.00–6.00)^e^	15	18.1	85	25.6			
High (> 6.00)^e^	16	19.3	80	24.1	1.0		
Generalized Obesity							
Obese	71	85.6	142	42.8	7.92	4.14–15.1	*p* < 0.001
Non-obese	12	14.4	190	57.2			
Central Obesity-							
Centrally obese	73	88.0	218	65.7	3.81	1.89–7.67	*p* < 0.001
Normal	10	12.0	114	34.3	1.0		
Exposure to passive smoking							
Yes	31	37.3	76	22.9	2.01	1.20–3.35	*p* = 0.01
No	52	62.7	256	77.1	1.0		
Hair Dye							
Ever used	29	34.9	145	43.7	0.69	0.42–1.14	*p* = 0.15
Never used	54	65.1	187	56.3	1.0		
Exposure to Electromagnetic Field							
Yes	16	19.3	73	22.0	0.85	0.46–1.55	*p* = 0.59
No	67	80.7	259	78.0	1.0		
Exposure to Pesticide/weedicide							
Direct exposure	12	14.5	15	4.5	2.50	1.17–5.32	*p* = 0.28
Indirect exposure	0	0.0	6	1.8			
No exposure	71	85.5	311	93.7	1.0		
Exposure to air pollution							
Yes	46	55.4	167	50.3	1.23	0.76–1.99	*P* = 0.40
No	37	44.6	165	49.7	1.0		
Total	83	100.0	332	100.0			

^a^Sub-optimal: Inadequate consumption of dietary fibre, inadequate consumption of anti-oxidants and over consumption of energy dense food

^b, c, d, e^Average and high levels of physical activity categories amalgamated for bivariate analysis

There was a higher proportion of hypertension (*p* = 0.02) and use of Losartan for ≥5 years (*p* = 0.03), use of Tamoxifen (*p* < 0.001), ever exposure of x-ray radiation (*p* < 0.001), past history of adenomyosis (*p* = 0.01) and family history of any type of cancer (*p* < 0.001) in the case group as compared with those of the control group (Table 4).

**Table 4:** Risk of Endometrial Carcinoma associated with Medical Conditions

Characteristics	Disease Status				OR	95% CI	*p* value
	Cases (*N* = 83)		Controls (*N* = 332)				
	n	%	n	%			
Duration of Diabetes Mellitus							
≥ 5 years	18	21.7	59	17.8	1.28	0.71–2.32	*p* = 0.41
< 5 years	65	78.3	273	82.2	1.0		
Duration of Hypertension							
≥ 5 years	25	30.1	62	18.7	1.88	1.10–3.23	*p* = 0.02
< 5 years	58	69.9	270	81.3	1.0		
Duration of Hyperlipidemia							
≥ 5 years	10	12.0	34	10.2	1.20	0.57–2.54	*p* = 0.63
< 5 years	73	88.0	298	89.8	1.0		
On long term medication							
≥ 5 years	34	41.0	102	30.7	1.56	0.95–2.56	*p* = 0.07
< 5 years	49	59.0	230	69.3	1.0		
Tamoxifen							
Yes	4	4.8	0	0.3	5.20	4.27–6.34	p < 0.001
No	79	95.2	332	99.7	1.0		
Exposure to any x-ray							
Ever exposed	63	75.9	185	55.7	2.50	1.45–4.33	*p* < 0.001
Never exposed	20	24.1	147	44.3	1.0		
Fibroid							
Yes	12	14.5	80	24.1	0.53	0.27–1.03	*p* = 0.06
No	71	85.5	252	75.9	1.0		
Endometriosis							
Yes	20	24.1	72	21.7	1.15	0.65–2.02	*p* = 0.63
No	63	75.9	260	78.3	1.0		
Adenomyosis							
Yes	8	9.6	74	22.3	0.37	0.17–0.81	*p* = 0.01
No	75	90.4	258	77.7	1.0		
PID^1^							
Yes	4	4.8	14	4.2	1.15	0.37–3.59	*p* = 0.81
No	79	95.2	318	95.8	1.0		
Vaginal Infection							
Yes	9	10.8	36	10.5	1.03	0.47–2.24	*p* = 0.93
No	74	89.2	296	89.5	1.0		
History of PCOD^2^							
Yes	8	9.6	17	5.1	1.98	0.82–4.75	*p* = 0.12
No	75	90.4	315	94.9	1.0		
Family history of any type of cancer							
Yes	31	37.3	19	5.7	9.82	5.17-	*p* < 0.001
No	52	62.7	313	94.3	1.0	18.66	
Total	83	100.0	332	100.0			

There were 10 variables retained in the final regression model of the 32 variables that were included. The sample size for the final regression model included 415 postmenopausal women with 83 cases and 415 controls. After adjusting the variables, having family history of any type of cancer (OR = 12.61, 95% CI:5.14–30.94), generalized obesity (OR = 11.85, 95% CI:5.12–27.43), never conceived (OR = 3.84, 95% CI:1.37–10.71), age more than 55 years (OR = 4.69, 95% CI:2.16–10.21), monthly family income ≤20,000 Sri Lankan Rupees (OR = 2.65, 95% CI:1.31–5.39), age at menarche ≤11 years (OR = 4.07, 95% CI:1.16–14.21), and low level household activities (OR = 2.82, 95% CI:1.34–5.92) were found to be associated with an increased risk of endometrial carcinoma (Table 5).

**Table 5:** Adjusted Odds Ratios for Independent risk factors of Endometrial Carcinoma

Independent risk factors	β	SE (β)	AOR^a^	95% CI for AOR^a^		*p* value
				Lower	Upper	
Having family history of any type of cancer	2.53	0.46	12.61	5.14	30.94	*p* < 0.001
Generalized obesity BMI ≥ 25 Kgm^−2^	2.47	0.43	11.85	5.12	27.43	*p* < 0.001
Never conceived	1.34	0.52	3.84	1.37	10.71	*p* = 0.01
Age more than 55 years	1.55	0.39	4.69	2.16	10.21	*p* < 0.001
Monthly family Income ≤ Rs.20,000	0.97	0.36	2.65	1.31	5.39	*p* = 0.007
Age at menarche ≤11 years	1.40	0.64	4.07	1.16	14.21	*p* = 0.03
Sub-optimal consumption of deep fried food	−1.74	0.49	0.17	0.06	0.46	*p* < 0.001
Low level household activities	1.03	0.38	2.82	1.34	5.92	*p* = 0.006
Central obesity> 80 cm	0.79	0.45	2.21	0.92	5.31	*p* = 0.07
Ever exposed to X-ray	0.61	0.38	1.85	0.88	3.87	*p* = 0.10
Constant	−6.20	0.80	.002			*p* < 0.001

^a^*AOR* Adjusted Odds Ratio

## Discussion

The independent risk factors of endometrial carcinoma among postmenopausal women were having family history of any type of cancer, generalized obesity, never conceived, sub-optimal consumption of deep fried food, low level household activities, age more than 55 years, monthly family income ≤ Sri Lankan Rupees 20,000 and age at menarche ≤11 years.

In the present study, family history of cancer among first degree relative had shown a strong association with endometrial carcinoma. Several studies had also assessed the association of family history of cancer among first degree relative with endometrial carcinoma and the results showed consistencies [18, 19] as well as inconsistencies [20] with the findings of the present study.

Another important finding of the present study was being generally obese increased the risk of developing endometrial carcinoma. This result is in line with the findings of two other studies [21, 22]. All these evidence in line with the present study reiterate the importance of maintaining BMI within the normal range recommended for women in preventing endometrial carcinoma and to be emphasized in health promotion programme for women in Sri Lanka.

The present study identified having history of never conception as an independent risk factor for endometrial carcinoma. This finding was in line with another study [23].

In the present study, sub-optimal consumption of deep fried food was an independent association with endometrial carcinoma. Contrary to the common belief ‘frequent consumption of deep fried food could cause cancer’, present study identified sub optimal consumption of deep fried food with a protective effect in developing endometrial carcinoma. The lifestyle changes among the cases due to the diagnosis of the disease would have influenced the validity of data. Several studies found that there was no significant association of dietary practices and developing endometrial carcinoma [24–26] while one study [27] showed a significant association with endometrial carcinoma with a protective effect in consumption of butter and coffee. Due to the differences of the tools and the methodologies used to assess the dietary practices in the above studies, meaningful comparison cannot be done.

Another independent risk factor for endometrial carcinoma was low level lifetime household activities. A study [28] revealed a reduced risk of endometrial carcinoma among women who spent 90 min or more of non-occupational physical activities compared to women who spent less than 30 min per day. Another study [29] also revealed a decreased risk of endometrial carcinoma associated with leisure time activities.

In the present study, age 55 years or more found to be a significant risk factor for endometrial carcinoma. The risk of developing endometrial carcinoma increases with age [30]. Though, age being a non-modifiable factor, this finding indicates that preventive interventions for endometrial carcinoma should be targeted at young women to prevent the development of the disease at the later ages in life.

Further, present study found that monthly family income of Sri Lankan Rupees 20,000 or less was an independent association with endometrial carcinoma. This could be due to various unhealthy practices related to low family income such as unhealthy dietary practices, and poor care seeking behaviour [31].

The present study identified menarcheal age of 11 years or less as an independent risk factor for endometrial carcinoma. The findings are consistent with the finding of another study [32].

Several studies reported that history of diabetes mellitus and hypertension are associated with increased risk of endometrial carcinoma [33–36]. In the current study, diabetes mellitus didn’t show any significant association though hypertension increased the risk of endometrial carcinoma in the bivariate analysis. This contradictory results could be explained in part by the method of assessment of the two conditions.

Generalizability of the results was ensured by selecting a representative sample of cases and controls from all government hospitals in the Western province with facilities to diagnose and treat the endometrial carcinoma patients.

A major limitation in case control study design is selection bias. Ideally, the controls should be selected from the community where the cases belonged, since the controls should be comparable to the source population of the cases. Though, it is unethical to carry out invasive procedures to healthy women to exclude any cancer status at the time of selection. For assessing the association of family history of any type of cancer and endometrial carcinoma, the patients diagnosed with EC with or suspected lynch syndrome should be excluded, as endometrial carcinoma - lynch syndrome relationship dilutes the effect of family history on endometrial carcinoma. In the present study, the influence of hereditary component in endometrial carcinoma was not assessed due to limited resource and time in the local setup. This was another limitation of the study. Selection of several common risk factors of endometrial carcinoma to calculate the sample size would result in lack of statistical power for other important risk factors with marginal effect size leading to wide confidence intervals due to the limited sample size. This is considered as a limitation of the study.

## Conclusions

Of the potential risk factors, age more than 55 years, monthly family income of Sri Lankan Rupees 20,000 or less, history of never conception, early menarche, having family history of any type of cancer, generalized obesity, sub-optimal consumption of deep fried food and low level household activities were the independent risk factors of endometrial carcinoma specific for Sri Lankan postmenopausal women. The importance of inculcating healthy dietary and physical activity practices since the childhood need to be advocated and addressed through public awareness programmes. Community level educational programmes on planning healthy meals at home should be organized to uplift the knowledge of women who are usually responsible in deciding main meal at home.

## Supplementary information

**Additional file 1.** Interviewer Administered Questionnaire for assessing Risk Factors for Endometrial Carcinoma.

**Additional file 2.** Food Frequency Questionnaire.

**Additional file 3.** Life Time Total Physical Activity Questionnaire.

## Data Availability

The datasets used and/or analysed during the current study are available from the corresponding author on reasonable request.

## Abbreviations

AOR: Adjusted Odds Ratios
BMI: Body Mass Index
CI: confidence intervals
EC: Endometrial cancer
HRT: Hormone Replacement Therapy
IQR: Interquartile Range
MET: Metabolic Equivalent of Task
OR: Odds Ratio
SPSS: Statistical Package for the Social Sciences
WHO: World Health Organization
