# Supplementary material for: Risk factors for endometrial carcinoma among postmenopausal women in Sri Lanka: a case control study
Source: BMC Public Health. 2019 Oct 28;19:1387. doi: 10.1186/s12889-019-7757-2 (PMC6816310; doi:10.1186/s12889-019-7757-2)
Supplement: Supplementary file 1 — Additional file 1. Interviewer Administered Questionnaire for assessing Risk Factors for Endometrial Carcinoma. [file 12889_2019_7757_MOESM1_ESM.docx]

**Questionnaire 1: Study to identify the risk factors of endometrial cancer among postmenopausal women in the Western province**

| Serial number: | \|  \|  \|  \|  \|  \|  \| \| --- \| --- \| --- \| --- \| --- \| --- \| | | | | | |  | | |  |  |  |
| --- | --- | --- | --- | --- | --- | --- | --- | --- | --- | --- | --- | --- | --- | --- | --- | --- | --- | --- |
|  |  | | | | | |  | | |  |  |  |
| Name of the interviewer: | | | | | | |  | | |  |  |  |
| Date of the interview : | | | | | | |  | | |  |  |  |
| **Section A : Basic information of the participant** | | | | | | | | | | |  |  |
|  |  | | | | | |  | | Hospital | |  | |
| Name: |  | | | | | |  | | Ward | |  | |
|  |  |  |  |  |  |  |  |  | BHT/Clinic No | |  | |
| Address: |  | | | | | | | | | | | |
| Contact number: | | | | | | |  | |  | |  |  |
| **Section B : Demographic and Socio-economic factor** | | | | | | | | |  | |  |  |
| 1. What is your age in completed years? | | | | | | | |  |  | |  |  |
| DOB |  |  |  |  |  |  |  | |  | |  |  |
|  | D | D | M | M | Y | Y |  | |  | |  |  |

2. Which ethnic group do you belong to?

| a. | Sinhala |  |
| --- | --- | --- |
| b. | Tamil |  |
| c. | Muslim |  |
| d. | Burger |  |
| e. | Other (specify) |  |

3. What is your religion?

| a. | Buddhist |  |
| --- | --- | --- |
| b. | Catholic/Christian |  |
| c. | Islam |  |
| d. | Hindu |  |
| e. | Other (specify) |  |

4. What is your highest level of education?

| a. | No formal education |  |
| --- | --- | --- |
| b. | Grade 1-5 |  |
| c. | Grade 6-10 |  |
| d. | Ordinary level completed |  |
| e. | Grade 11-12 |  |
| f. | Advanced level completed |  |
| g. | Technical/vocational/diploma |  |
| h. | University and higher |  |

5. Are you currently employed/had been employed?

| a. | Currently employed |  |
| --- | --- | --- |
| b. | Had been employed |  |
| c. | Never employed |  |

6. What is your monthly family income?

| a. | < Rs.10,000 |  |
| --- | --- | --- |
| b. | Rs.10,001-20,000 |  |
| c. | Rs.20,001-30,000 |  |
| d. | More than 30,000 |  |

**Section C: Risk factors**

1. **Hormonal and Reproductive**

7. What is your current marital status?

| a. | Unmarried |  |
| --- | --- | --- |
| b. | Married |  |
| c. | Divorced/separated |  |
| d. | Widowed |  |
| e. | Other (specify) |  |

8. Have you ever become pregnant? (If no, go to Q 10)

| a. | Yes |  |
| --- | --- | --- |
| b. | No |  |

9. If yes (Q 8), information regarding past obstetric history.

| Pregnancy | Age at pregnancy | Outcome* | POA at outcome |
| --- | --- | --- | --- |
|  |  |  |  |
|  |  |  |  |
|  |  |  |  |
|  |  |  |  |

*LB(Live birth), SB(Still birth), SA(spontaneous abortion), IA(Induced

abortion),EP(Ectopic pregnancy)

10. At what age/approximate age did you attain menarche? …………………………….

If you are not sure about exact age, please select the age range.

| a. | 11 years or less |  |
| --- | --- | --- |
| b. | 12-15 years |  |
| c. | More than 15 years |  |

11. At what age/approximate age did you stop your menstruation? ……………………………..

If you are not sure about exact age, please select the age range.

| a. | Less than 45 years |  |
| --- | --- | --- |
| b. | 45-50 years |  |
| c. | 51-55 years |  |
| d. | More than 55 years |  |

12. Have you ever used Hormone Replacement Therapy (HRT)?

| a. | Yes |  | If yes, go to Q 13 |
| --- | --- | --- | --- |
| b. | No |  | If no, go to Q 17 |

13. What is the total duration of use of HRT?

| a. | < 1 year |  |
| --- | --- | --- |
| b. | 1-5 years |  |
| c. | > 5 years |  |

14. What was the indication to start HRT?

| a. | To relieve postmenopausal symptoms |  |
| --- | --- | --- |
| b. | To replace hormone at premature ovarian failure |  |
| c. | As a medication in other illnesses |  |

15. What was the age at initiation of HRT?

| a. | < 45 years |  |
| --- | --- | --- |
| b. | 45-50 years |  |
| c. | 51-55 years |  |
| d. | >55 years |  |

16. What was the type of HTR used?

| a. | Oestrogen only |  |
| --- | --- | --- |
| b. | Combined therapy (Oestrogen and progesterone) |  |

17. Have you ever used hormonal contraceptives?

| a. | Yes |  | Methods: OCP (Oral contraceptive method),DMPA (Depo-Provera),IMP (Implant) |
| --- | --- | --- | --- |
| b. | No |  |  |

1. **Life style related factors**
   1. **Diet**

18. Food frequency questionnaire (FFQ) to assess individual dietary practice

- 1. **Physical activity**

19. Lifetime total physical activity questionnaire (LTPAQ)

- 1. **Personal factors**

20. Have you ever consumed alcohol?

| a. | Never tried |  |
| --- | --- | --- |
| b. | Ever used |  |
| c. | Currently using |  |

21.If ever used/ currently using (Q 20), how frequently are you consuming alcohol or had consumed alcohol in the past?

| a. | Daily |  |
| --- | --- | --- |
| b. | Weekly |  |
| c. | Monthly |  |
| d. | Occasional |  |

22. What is the usual amount of alcohol consumed per day or had consumed per day in the past?

| a. | Less than ¼ bottle |  |
| --- | --- | --- |
| b. | ¼ to ½ bottle |  |
| c. | More than ½ bottle |  |

23. Smoking status

| a. | Never smoked/ No passive smoking |  |  |
| --- | --- | --- | --- |
| b. | Never smoked/with passive smoking |  | Go to Q 24 |
| c. | Former smoker |  |  |
| d. | Current smoker |  |  |

24. If you had never smoked, but if you are exposed to passive smoking, the duration of exposure to passive smoking per day

| a. | < 1 hour |  |
| --- | --- | --- |
| b. | 1-2 hours |  |
| c. | 2-3 hours |  |
| d. | > 3 hours |  |

25. Have you ever used hair dyes?

| a. | Yes |  |
| --- | --- | --- |
| b. | No |  |

26. If yes (Q 25), What was your usual pattern of using hair dyes?

| a. | Weekly |  |
| --- | --- | --- |
| b. | Monthly |  |
| c. | Occasionally or Never |  |

1. **Environmental related factors**
   1. **External environment**

27. Are there any high tension wires in the vicinity of your home?

| a. | Yes |  |
| --- | --- | --- |
| b. | No |  |

28. If yes (Q27), can you give the approximate distance?

| a. | < 50m |  |
| --- | --- | --- |
| b. | 50 -100 |  |
| c. | > 100m |  |

29. Are there any mobile transmission sites in the vicinity of your home?

| a. | Yes |  |
| --- | --- | --- |
| b. | No |  |

30. If yes (Q29), can you give the approximate distance?

| a. | < 50m |  |
| --- | --- | --- |
| b. | 50 -100 |  |
| c. | > 100m |  |

31. What is the distance to your house from the public road where buses travel?

| a. | < 50m |  |
| --- | --- | --- |
| b. | 50 -100 |  |
| c. | > 100m |  |

32. Are there any large industries in Vicinity?

| a. | Yes |  |
| --- | --- | --- |
| b. | No |  |

33. If yes (Q32), what is the approximate distance from your home?

| a. | < 500m |  |
| --- | --- | --- |
| b. | 500m to 1km |  |
| c. | > 1km |  |

- 1. **Exposure to pesticide**

34. Have you ever exposed to pesticide/ weedicide?

| a. | Yes |  |
| --- | --- | --- |
| b. | No |  |

35. If yes (Q 34), how have you been exposed to agrochemicals?

| a. | Accidental or intentional ingestion |  |
| --- | --- | --- |
| b. | Exposed directly when working in a farm or paddy field(at least 6 months) |  |
| c. | Exposed at household level (at least 6 months) |  |
| d. | Living in close proximity to environment using agrochemicals (at least 6 months) |  |

| 1. **Co-morbid factors** |
| --- |
| - 1. **Medical information**   36. Are you suffering from any long term disease condition?   \| a. \| Yes \|  \| \| --- \| --- \| --- \| \| b. \| No \|  \|     37. If yes (Q 36), description about the disease? (confirmed with written evidence)   \| Disease \| Duration(years) \| \| --- \| --- \| \|  \|  \| \|  \|  \| \|  \|  \| \|  \|  \| |

38. Are you currently taking any drugs on long term basis?

| a. | Yes |  |
| --- | --- | --- |
| b. | No |  |

39. If yes (Q 38), description about the drugs (confirmed with prescription)

| Name of the drug | Duration | Dosage |
| --- | --- | --- |
|  |  |  |
|  |  |  |
|  |  |  |
|  |  |  |
|  |  |  |
|  |  |  |
|  |  |  |

40. Have you ever treated with drug Tamoxifen?

| a. | Yes |  |
| --- | --- | --- |
| b. | No |  |

41. Were you ever been X-rayed?

| a. | Yes |  |
| --- | --- | --- |
| b. | No |  |

42. Are you suffering from any other disease related to reproductive tract (confirm with medical records)?

| a. | PCOD (irregular periods, acne, excessive facial and body hair growth) |  |
| --- | --- | --- |
| b. | Endometriosis |  |
| c. | Fibroids |  |
| d. | Adenomyosis |  |
| e. | Vulvo-vaginitis |  |
| f. | Pelvic inflammatory disease |  |

1. **Genetic factors/ Family history**

43. Has any one of your immediate family members (parents, children, brothers and sisters) suffered/ are suffering from the same illness you are suffering or any other illness related to cancer?

| a. | Yes |  |
| --- | --- | --- |
| b. | No |  |

44. If Yes (Q 43), what is your relationship with the above mentioned family member?

| a. | Father |  |
| --- | --- | --- |
| b. | Mother |  |
| c. | Brother/ Sister |  |
| d. | Children |  |

45. What is his/her diagnosis?

|  |
| --- |

46. What was his/her age of the diagnosis of the illness?

|  |
| --- |

**Thank you for answering the above questions.**
