# Supplementary material for: Risk factors for endometrial carcinoma among postmenopausal women in Sri Lanka: a case control study
Source: BMC Public Health. 2019 Oct 28;19:1387. doi: 10.1186/s12889-019-7757-2 (PMC6816310; doi:10.1186/s12889-019-7757-2)
Supplement: Supplementary file 2 — Additional file 2. Food Frequency Questionnaire. [file 12889_2019_7757_MOESM2_ESM.docx]

**Food Frequency Questionnaire to assess individual dietary practices**

The following questions are about your dietary practices.

1. What is your daily food consumption pattern?

Indicate by the number of meals and their portion size that you normally consume during one of your usual weeks. (Show her the visual guide that indicate the portion sizes of different types of food and explain how to gauge the size of their meals).

| a. | Large size meal | 1 |
| --- | --- | --- |
| b. | Medium size meal | 2 |
| c. | Small size meal | 3 |

1. Have you been following a diet prescribed by a dietician?

| a. | Yes | 1 |
| --- | --- | --- |
| b. | No | 2 |

**Food frequency questionnaire- before getting the disease**

Now think about the normal dietary habits before getting the disease/during past one year. Tell me how often on average you have eaten the following food item/s. Remember to include things that you cook with. These questions are not intended to assess your total diet, and therefore may not find all the foods you eat. When answering these questions, please make use of the guide given to you for identifying a medium portion size for each food types. Ignore that were taken in lesser amounts than indicated by the visual food guide. Each food group has 5 responses to indicate how often on average you have eaten food items. They are as:

| a. | Less than one per week | 1 |
| --- | --- | --- |
| b. | Once per week | 2 |
| c. | 2-4 times per week | 3 |
| d. | More than 4 times per week or nearly daily | 4 |
| e. | Twice or more per day | 5 |

| 1. | Green leaves (Kankun, spinach, gotukola, mukunuwenna, sarana), dark green leafy vegetables (leeks, leaves of beet, raddish, cabbage), Katurumurunga |  |
| --- | --- | --- |
| 2. | Starchy vegetables (Jack, breadfruit, manioc, sweet potato, potatoes, pumpkin) |  |
| 3. | Other vegetables (brinjals, ladies fingers, beans, pathola, wetakolu, beet, nokol) |  |
| 4. | Citrus fruits (oranges, grapefruit, lime, lemon, jamanaran, naran) |  |
| 5. | Yellow fruits (mangos, papaw) |  |
| 6. | Other fruits (apples, pears, bananas, grapes, melon, wood apple) |  |
| 7. | Whole milk products (fresh milk, sterilized milk, milk powder, sweetened milk) |  |
| 8. | Main dairy products (ice cream, yoghurt, curd) |  |
| 9. | Other dairy products (cheese, butter, ghee) |  |
| 10. | Low fat dairy products (Nonfat milk, Nonfat yoghurt) |  |
| 11. | Hard margarine (Which are hard at a low temperature eg: Astra) |  |
| 12. | Soft margarine (which are soft at a low temperature eg: Flora) |  |
| 13. | Whole eggs/ egg based products (caramel pudding, watalappan) |  |
| 14. | Whole grain products (brown rice, red rice, flour based food, kurakkan wheat products, whole grain bread/cereals/buns,bran) |  |
| 15. | Pulses as main dish (green grams, gram, kaupi, ulundu flour based food) |  |
| 16. | Red meat (beef, mutton, pork), chicken with skin |  |
| 17. | Processed meat (sausages, bacon, meat balls, hot dogs, ham burgers) |  |
| 18. | Fish/ Sea food (not fried) |  |
| 19. | Commercially baked food-oven baked food (pastries, pies, pizza, muffins, savoury buns) |  |
| 20. | Rotti (pol rotti, stuffed rotti, khottu rotti) |  |
| 21. | Deep fried food- savoury food(rolls, cutlets, patties, wade),potato chips, deep fried meat or fish, deep fried snacks (eg: bites, kadju) |  |
| 22. | Sugar- sweetened beverages (sweetened drinks, fizzy drinks, tea with more than 2 teaspoons) |  |
| 23. | Soy products (soya meat) |  |
