# Supplementary material for: Risk factors for endometrial carcinoma among postmenopausal women in Sri Lanka: a case control study
Source: BMC Public Health. 2019 Oct 28;19:1387. doi: 10.1186/s12889-019-7757-2 (PMC6816310; doi:10.1186/s12889-019-7757-2)
Supplement: Supplementary file 3 — Additional file 3. Life Time Total Physical Activity Questionnaire. [file 12889_2019_7757_MOESM3_ESM.docx]

**Lifetime Total Physical Activity Questionnaire (LTPAQ)**

**Occupational and Volunteer activities**

Starting with your occupational activities, please tell me what jobs (paid or volunteer) you have done for at least 8 hours per week for 4 months of the year (128 hours total per year or 2.5 hours per week per year) over your lifetime starting with your first job.

Please tell me about each job that you had. I need to know how old you were when you started and stopped working at each job and the number of months per year, days per week, hours per day that you worked at each job. Finally, I need to know what kind of physical effort you had for each job. Please choose one intensity level from the list on this separate page that defines each level.

| No. | Job Title | Description of occupational activity | Age started | Age ended | No. of Mos/Yr | No.of Days/Wk | Time/Day | | Intensity of activity  (1,2,3,4) | Did you ever walk or bike to this job? | Which ones did you normally do?(check all that apply) | No of Mos/Yr | No.of Days/Wk | Time/Day | |
| --- | --- | --- | --- | --- | --- | --- | --- | --- | --- | --- | --- | --- | --- | --- | --- |
| 1. |  |  |  |  |  |  |  |  |  | - Yes - No (next job) - Ref (next job) - DK(next job) | - Walk - Bike - Other------ - Ref(next job) - DK(next job) |  |  |  |  |
| 2. |  |  |  |  |  |  |  |  |  | - Yes - No (next job) | - Walk - Bike - Other |  |  |  |  |

**Household Activities**

Now I am going to ask you to tell me about your patterns of household and gardening activities over your lifetime. Again, we will start with your past activity and then continue up to your reference year. Please include only those activities that you have done at least **7 hours per week 4 months** of the year (112 hours total per year or 2.14 hours per week per year).

It may help you to consider what a typical day or week was for you. Then think about how many hours of household, gardening, yard work or do-it yourself jobs around your home that you did in a typical day or week. For seasonal activities, such as gardening, you can report those separately from all other household activities that are done all year. Seated activities (such as sewing or paying bills) are not included. **Childcare** and **housework** is included.

| No. | Age started | Age ended | Number of Months/Yr | Number of Days/Wk | Time per day  Hrs Mins | | Hours per day spent in activities that were in category:  2 3 4 | | |
| --- | --- | --- | --- | --- | --- | --- | --- | --- | --- |
| 1. |  |  |  |  |  |  |  |  |  |
| 2. |  |  |  |  |  |  |  |  |  |
| 3. |  |  |  |  |  |  |  |  |  |

**Exercise and Sports Activities**

Now I would like to know all your exercise or sports activities that you did during your lifetime starting with your childhood and continuing to your reference year. Please report the activities that you have done at least 2 hours per week for 4 months of the year (32 hours total per year or 40 minutes per week per year).

Please tell us what exercise and sports activities you have done at least 10 times during your lifetime. Besides, sports and exercise, we are also interested in knowing whether you walked, biked or ran to school. If you have done this, please report all the information as for the other sports activities. Please begin by telling me the activities that you did during your school years including your physical education (gym) classes.

| No. | Description of  Exercise / sports activity | Code | Age started | Age ended | Frequency of activity | | | | Time per activity  Hrs Min | | Intensity of activity(2,3,4) |
| --- | --- | --- | --- | --- | --- | --- | --- | --- | --- | --- | --- |
|  |  |  |  |  | Day | Week | Month | Year |  |  |  |
| 1. |  |  |  |  |  |  |  |  |  |  |  |
| 2. |  |  |  |  |  |  |  |  |  |  |  |
| 3. |  |  |  |  |  |  |  |  |  |  |  |
| 4. |  |  |  |  |  |  |  |  |  |  |  |
